# Taiji as a Health-Promoting Mind–Body Exercise: Linking Movement, Learning, and Cognitive Health

**DOI:** 10.21315/mjms-05-2026-363

**Published:** 2026-06-30

**Authors:** Sze Kiat Sim

**Affiliations:** Department of Surgery, Faculty of Medicine and Health Sciences, Universiti Malaysia Sarawak, 94300 Kota Samarahan, Sarawak, Malaysia

**Keywords:** Taiji, Tai Chi, mind-body exercise, cognitive health, balance control

## Abstract

Taiji is a traditional Chinese mind–body exercise that is increasingly recognised for its potential benefits for cognitive health. Although it originated as a martial art, Taiji is now widely practised as a low-cost and culturally acceptable intervention for promoting healthy ageing and supporting cognitive resilience. Previous clinical and neuroimaging studies have demonstrated improvements in cognitive functions, including memory, attention, and executive functions, alongside structural and functional brain changes following regular Taiji practice. However, the underlying mechanisms responsible for these benefits remain incompletely understood. This commentary reviews the historical background of Taiji, summarises current evidence regarding its cognitive benefits, and highlights potential neurobiological mechanisms, including sensorimotor integration through balance control, repeated activation of proprioceptive pathways, and the modulation of thalamic and cortical networks. Comparisons with conventional forms of physical activity are also discussed. Given Malaysia’s rapidly ageing population, integrating Taiji into public health promotion and preventive healthcare strategies may support healthy ageing and therefore warrants further research and policy consideration.

## Introduction

Taiji, also known as Tai Chi, is a traditional Chinese mind–body practice that has evolved from martial arts into a widely accessible form of exercise. The term “Taiji” is derived from *Hanyu Pinyin*, whereas “Tai Chi” is a commonly used transliteration in Western literature (1). Despite uncertainty regarding the origins of Taiji, two major historical narratives are commonly cited. One traditional narrative attributes the origin of Taiji to Zhang Sanfeng, a legendary Taoist figure believed to have lived during the late Yuan and early Ming dynasties (2, 3). According to legend, his observation of a fight between a crane and a snake is said to have inspired the principle of “using softness to overcome hardness,” which is associated with the Yin–Yang concept. Another narrative credits Chen Wangting, a military officer from Chen Village in Henan Province who lived during the late Ming and early Qing dynasties, with formalising the Chen style, from which the Yang, Wu/Hao, Wu, and Sun styles later developed (4).

Despite stylistic diversity, practitioners emphasise a shared identity under the principle of “Taiji as One Family.” In recognition of its cultural significance, Taiji was inscribed on the United Nations Educational, Scientific and Cultural Organisation’s Representative List of Intangible Cultural Heritage of Humanity in 2020 (5). Although traditionally practised as a martial art, Taiji is recognised today as a moderate-intensity exercise with demonstrated health benefits, including cardiovascular conditioning and improved postural stability (6). Practising Taiji for approximately 50 minutes can elevate heart rate to approximately 68% of age-predicted maximal heart rate (HRmax), which is comparable to 50 minutes of brisk walking (6).

## Evidence for Cognitive Benefits

Over the past two decades, researchers have documented a wide range of health benefits associated with Taiji in older adults, including enhanced relaxation, improved cardiovascular fitness, improved blood pressure regulation, greater postural stability, and reduced risk of falls (1, 6–7). These outcomes are largely attributable to the moderate intensity of Taiji movements, which simultaneously engage multiple muscle groups. Moreover, the distinctive walking patterns, characterised by weight-bearing and shifting movements, help strengthen the hip and thigh muscles while promoting improved motor coordination.

Although Taiji is often promoted for improving musculoskeletal and cardiovascular outcomes, a growing body of literature has identified its potential cognitive benefits. Clinical and community-based studies suggest that regular practice is associated with improvements in executive functions, memory, and attention among older adults (8). Furthermore, Taiji has been associated with a reduced risk of mild cognitive impairment and dementia, and has been demonstrated to enhance working memory and processing speed in healthy adults (9). An exploratory study also noted that Taiji improved response and attentional inhibition, as measured using the antisaccade and visual search tasks with eye-tracking assessment (10).

Neuroimaging studies have provided further evidence to support these findings. Functional magnetic resonance imaging (fMRI) investigations have confirmed increased functional connectivity and greater grey matter volume in brain regions linked to learning and memory, attention, and executive functions, particularly the hippocampus, amygdala, and cingulate cortex within the limbic system, after 12 weeks of Taiji practice (11–12). These findings indicate that the cognitive benefits of Taiji are accompanied by measurable structural and functional neural adaptations. However, the mechanisms through which Taiji practice leads to these changes remain insufficiently understood.

## Potential Mechanisms

Taiji is unique in that it integrates motor, sensory, and cognitive components. The potential mechanisms underlying the cognitive benefits of Taiji are not yet well established (Figure 1). Taiji practice involves learning, memorising, and recalling movement sequences which engage hippocampal circuits and may enhance synaptic plasticity (13). Motor control, movement coordination and postural balance are also essential for the smooth execution of Taiji routines. These functions rely heavily on proprioceptive inputs from both upper and lower limbs. Proprioceptors, including muscle spindles and Golgi tendon organs, transmit signals via the dorsal column-medial lemniscus pathway to the thalamus, which then projects to the sensory cortex of the cerebrum. The thalamus, an integral component of the limbic system, plays a central role in emotion, memory, and motivation. With regular Taiji practice, perceptual and proprioceptive abilities are progressively enhanced. As the thalamus serves as a relay centre for both proprioceptive pathways and limbic circuits, proprioceptive stimulation may concurrently activate the hippocampus and amygdala (Figure 2). Through repeated training, Taiji may sustain thalamic activity and reinforce connectivity across neural networks that underpin learning and memory. This mechanistic framework is consistent with neuroimaging findings demonstrating increased regional brain activity and greater grey matter thickness in key brain regions involved in learning, memory, attention and executive functions following Taiji interventions (11–12).

## Comparison with Brisk Walking

Brisk walking, a moderate-intensity exercise, is widely recommended for cardiovascular and metabolic health. However, comparative studies have suggested that the neuroplastic changes detected using fMRI are significantly pronounced among Taiji practitioners (11). This distinction highlights the unique characteristics of Taiji, particularly its integration of physical movement, mindfulness, and controlled postural balance. Collectively, these elements may exert synergistic effects on cognition (8).

Current evidence suggests that practising Taiji three times per week for approximately one hour per session over 12 weeks can produce measurable cognitive benefit (14). Nevertheless, large-scale, longitudinal randomised controlled trials incorporating neuroimaging and biomarker outcomes are necessary to strengthen causal inferences.

## Implications for Malaysia

Malaysia is experiencing a demographic shift with a rapidly ageing population and a corresponding increase in the prevalence of dementia. Therefore, low-cost, culturally resonant, and accessible preventive strategies are urgently needed. Taiji offers a promising avenue, requiring minimal equipment, and can be practised individually or in groups, resonating with the cultural traditions across Asia.

Integrating Taiji into community health programmes, senior centres, and rehabilitation services could significantly contribute to promoting healthy ageing. Given its emerging neurobiological plausibility, Taiji should be considered not only as a form of physical activity but also as a modality for cognitive training.

## Conclusion

Taiji extends beyond a graceful martial art, functioning as a neurocognitive exercise with the potential to support brain health and enhance cognitive functions. Evidence suggests that Taiji practice strengthens memory, attention, and executive functions, which are underpinned by measurable neuroplastic changes. Compared with conventional aerobic exercise, the multidimensional integration of physical, cognitive, and mindful elements makes Taiji uniquely suited to support cognitive resilience.

As the population ages, particularly in Malaysia and across Asia, Taiji represents a practical and accessible strategy for supporting healthy ageing and reducing the risk of dementia. Integrating Taiji into community and healthcare settings may make a meaningful contribution to public health outcomes.

## Figures and Tables

**Figure 1:**
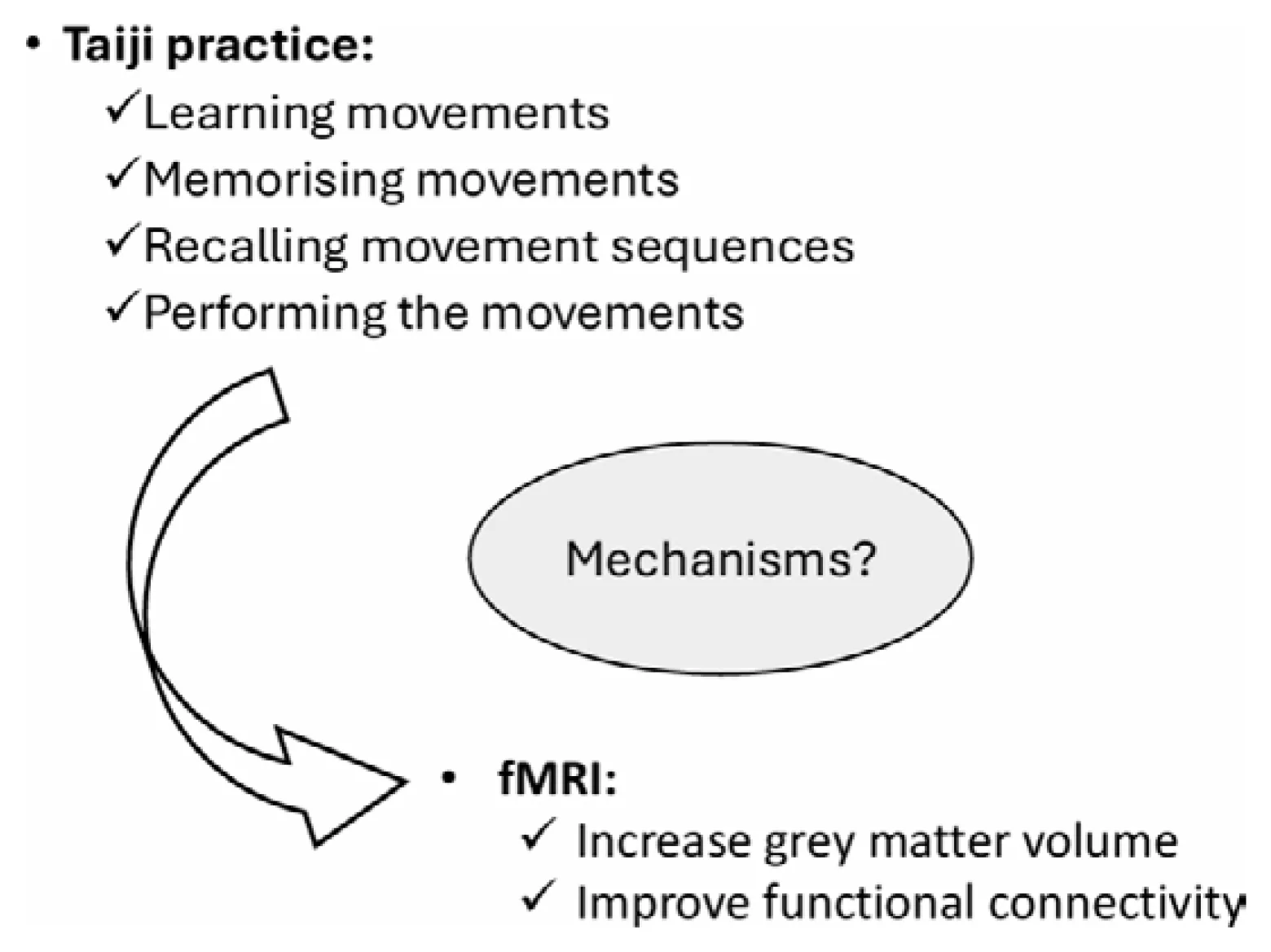
Effects of Taiji on brain health. Imaging studies have demonstrated structural and functional changes in brain regions associated with learning, memory, attention, and executive functions. However, the underlying mechanisms of these changes remain unclear.

**Figure 2:**
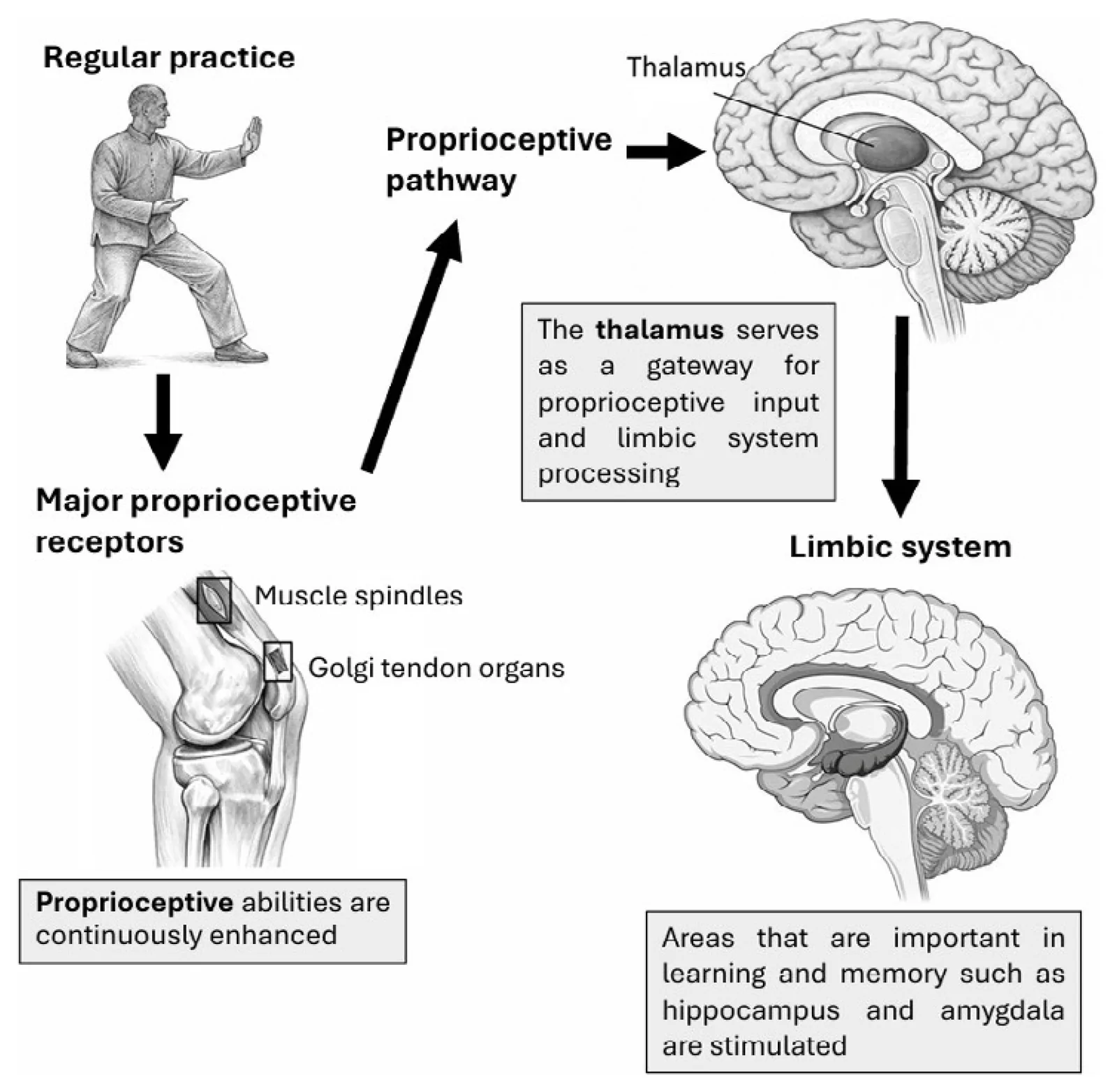
Mechanistic framework. Regular Taiji practice enhances proprioceptive activity. The thalamus, which serves as a relay centre for both proprioceptive pathways and limbic circuits, may facilitate concurrent activation of the hippocampus and amygdala through proprioceptive stimulation.
